# Determinants of health literacy in the general population: results of the Catalan health survey

**DOI:** 10.1186/s12889-019-7381-1

**Published:** 2019-08-16

**Authors:** Oriol Garcia-Codina, Dolors Juvinyà-Canal, Paloma Amil-Bujan, Carmen Bertran-Noguer, María Asunción González-Mestre, Eulàlia Masachs-Fatjo, Sebastià J. Santaeugènia, Pilar Magrinyà-Rull, Esteve Saltó-Cerezuela

**Affiliations:** 1 Health Planning General Directorate, Ministry of Health, Government of Catalonia, Barcelona, Spain; 2 Research Group Health and Health Care, University of Girona, Plaça de Sant Domènec, 3, 17004 Girona, Spain; 3 Chronicity Prevention and Care Programme, Health Planning General Directorate, Ministry of Health, Government of Catalonia, Barcelona, Spain; 4 Central Catalonia Chronicity Research Group (C3RG), Centre for Health and Social Care Research (CESS), Universitat de Vic – University of Vic-Central University of Catalonia (UVIC-UCC), Vic, Spain; 5 Chronic Care Program, Ministry of Health, Generalitat de Catalunya, Catalonia, Spain; 6 Public Health Agency. Ministry of Health, Government of Catalonia, Barcelona, Spain

**Keywords:** Health literacy, HLS-EU-Q16, Catalonia, Person-centered, Health care

## Abstract

**Background:**

Health Literacy (HL) is the knowledge and competence to access, understand, appraise, and apply health information for health judgment. We analyze for the first time HL level of Catalonia’s population. Our objective was to assess HL of population in our area and to identify social determinants of HL in order to improve the strategies of the Healthcare Plan, aimed at establishing a person-centered system and reducing social inequalities in health.

**Methods:**

This was a cross-sectional study based on the Health Survey for Catalonia (ESCA, *Enquesta de Salut de Catalunya*), which included the 16 items of the European Health Literacy Survey Questionnaire (HLS-EU-Q16). The statements in the questionnaire cover three different health literacy domains: Health Care, Disease Prevention, and Health Promotion. HL was categorized in three levels: Sufficient, Problematic and Inadequate. Chi-square tests were performed to compare the percentages of subjects with adequate or inadequate HL across sociodemographic and health-related variables. Variables showing significant differences were included in a stepwise logistic regression to predict inadequate HL level.

**Results:**

The questionnaire was administered to 2433 subjects aged between 15 and 98 years old (mean of 45.9 years, SD 18.0). Overall, 2059 subjects (84.6%) showed sufficient HL, 250 (10.3%) inadequate HL, and 124 (5.1%) problematic HL, with no significant differences between men and women (*p* = 0.070). A logistic regression analysis showed that low health literacy is associated with a lower level of education (OR 2.08, CI 95% 1.32–3.28, *p* = 0.002), low socioeconomic status (OR 2.11, CI 95% 1.42–3.15, *p* <  0.001) and a physical limitation to perform everyday activities (OR 2.50, CI 95% 1.34–4.66, *p* = 0.004). We also found a more modest association with low physical activity, having a self-perceived chronic disorder and performing preventive activities.

**Conclusions:**

Catalonia has a high percentage of subjects with sufficient HL. Education level, socioeconomic status and physical limitations were the factors with the strongest contribution to inadequate or problematic health literacy. Although these results are likely to be country-specific, the factors identified will allow policymakers of areas with similar socioeconomic profiles to identify groups with high risk of problematic or inadequate HL, which is essential for a successful patient-centered model of care.

## Background

One of the priorities of the World Health Organization (WHO) is to develop integrated and people-centered health services that allow reducing health costs and improve the quality of life. This makes it necessary to establish strategies to empower patients and increase their engagement in health decision making. Patients’ access to and understanding of health information becomes, therefore, essential [1, 2].

In recent years, the concept of Health Literacy (HL) has gained increasing attention in Public Health research as well as in health services reform processes, and now it is considered one of the essential factors and determinants of individual health and health service use. HL is an evolving concept, which has expanded from a simple understanding of health information to a comprehensive meaning of health aimed at empowering citizens for healthy living [3, 4]. After a systematic literature review of existing HL definitions and models, the European Health Literacy Consortium defined HL as “the knowledge, motivation and competence to access, understand, appraise, and apply health information in order to make judgments and take decisions in everyday life concerning health care, disease prevention and health promotion to maintain or improve quality of life throughout the course of life” [4]. This definition integrates three health relevant areas: health care, disease prevention, and health promotion [1].

Various tools have been proposed for measuring HL in the population [5]: designed either to assess specific HL aspects [6–10] (and often applied in clinical settings [11, 12]) or to approach HL in a comprehensive way [13]. In 2009, a consortium of nine organizations from eight EU member states designed the European Health Literacy Survey (HLS-EU) to apply it in the general population. The HLS-EU Questionnaire includes 47 items and it has been validated on a large, cross-national sample of EU citizens according to the well-established Eurobarometer methodology [14]. A shortened version with 16 items (HLS-EU-Q16) was developed to reduce completion time and difficulty and, therefore, make it easier to apply in the general population [15, 16]. In recent years, HLS-EU-Q16 has been used in various studies with uneven results [12, 17–23], although most focused on specific populations rather than the overall population. The psychometric properties of the HLS-EU-Q16 have been recently assessed in a neighbor region in Spain [24].

In Spain, the scope of action of health policies is regional, which allows implementing concrete actions in concrete populations. In Catalonia, a region in the northeast of Spain with 7.5 million people, the priorities of the Health Plan for Catalonia (HPC) are the establishment of a person-centered system and the reduction of social inequalities in health. The diagnosis of the initial scenario is, therefore, mainstay for developing and improving these strategies, and it must consider both the magnitude of the problem and its distribution according to social determinants in health. In Catalonia, a health survey (ESCA) is conducted every year using home interviews with the aim of providing relevant health information about the population and guiding the policy assessments outlined in the HPC [25]. ESCA gathers information on health status, lifestyles and the use of health services of the population of Catalonia. Taking advantage of the usability of a short questionnaire, the HLS-EU-Q16 was included in ESCA in 2014 to also gather information on the HL level of the general population. The objective of this study was to identify social and health-related determinants of HL in the general population in order to improve the person-centered health care services.

## Methods

### Study design and participants

This was a cross-sectional study based on the ESCA 2014 survey (a general questionnaire for subjects aged ≥15 years), which included the 16 items of HLS-EU-Q16. ESCA is an official survey performed by the Catalan Government (Health Ministry, Directorate General of Health Planning and Research) and by the Statistical Institute of Catalonia. The survey is administered every year to non-institutionalized male and female subjects aged 15 years old and over who are selected based on a multistage probability sampling, including all seven health regions defined according to geographical, socioeconomic and demographic characteristics. The sample is stratified by age, gender, municipality size, and health region. To maximize the number of respondents, ten substitutes with a matching age, gender, municipality (or nearby municipalities), and health region were assigned to each subject. Interviews were performed between January 13, 2014 and January 9, 2015 by *IPSOS Operaciones SA* using computer-assisted personal interviewing. Face-to-face home interviews were conducted by well-trained professionals.

The administration of the ESCA survey was approved by the Consultants’ Committee of Confidential Information Management (CATIC) from the Catalan Health Department, according to the Helsinki Declaration (revised in 2000). The anonymized results of the complete survey are publicly available at the Catalan Government web site [25].

### Measurements

The ESCA survey consists of nearly 500 questions addressing socioeconomic characteristics, health status, health-related behaviors, and the use of healthcare resources. For the purpose of this study, the following variables were analyzed: gender, age, employment status, education level, smoking, alcohol consumption, physical activity, health coverage, preventive activities, self-medication, weight, self-perceived health, visits to healthcare professionals, self-perceived chronic disorders, comorbidities, and physical limitations to perform everyday activities (Table 1).

**Table 1:** Variables analyzed

Variable	Category
Socio-demographic characteristics of study subjects	
Gender	Men
	Women
Age	15–44 years
	45–64 years
	65–74 years
	≥75 years
Employment status	Employed
	Unemployed
	Unpaid work
	Retiree/Permanently disabled
	Other
Socioeconomic status^a^	High (groups I and II)
	Middle (groups III and IVa)
	Low (groups IVb and V)
Education	Primary or non-regulated education
	Secondary education
	College/University/Post-graduate
Health behavior of study subjects	
Smoking	Smoker
	Former smoker
	Non-smoker
Alcohol consumption^b^	Non-drinker
	Light drinker
	Heavy drinker
Physical activity^c^	Healthy (high and moderate physical activity)
	Non-healthy (low physical activity)
Health coverage	Only public health coverage
	Public and private health coverage
	Only private health coverage
	No health insurance
Preventive activities^d^	Yes
	No
Self-medication^e^	Yes
	No
Self-consumption of dietary/ homeopathic products	Yes
	No
Subject-perceived health status	
BMI (Kg/m^2^)	Underweight (< 18,5)
	Normal Weight (≥18,5 a < 25)
	Overweight (≥25 a < 30)
	Obese (≥30)
Perceived general health^f^	Good health
	Bad health
Visits to the healthcare professional in the last 12 months	Family doctor
	Sexual and reproductive healthcare professional
	Mental health professional
	Other specialists
	Nurse
	Social worker
	Pharmacists and other community health agents
Medical consultation last year (at least once)	Yes
	No
Self-perceived chronic disorder	Yes
	No
Comorbidities^g^	Yes
	No
Mental health disorders	Yes
	No
Physical limitation to perform everyday activities	Yes, severely limited
	Yes, limited but not severely
	Not limited

*BMI* body mass index

^a^ Socioeconomic status was determined based on occupation the household head classified according to the Spanish adaptation of the British Registrar General’s classification [26, 27]

^b^Drinking behaviour was categorized according to the subject’s alcohol consumption habits as Non-Drinkers (subjects who have not consumed alcohol in the last 12 months), Light Drinkers, and Heavy Drinkers (men who drink more than 28 standard drinks per week, women who drink more than 17 standard drinks per week, or subjects who drink more than five consecutive alcoholic beverages once a month, regardless of sex). (In Spain, one standard drink contains 10 g of ethanol.) [28]

^c^Physical activity was categorized as Healthy and Unhealthy according to an adaptation of the International Physical Activity Questionnaire (IPAQ) short form [25, 29]

^d^Preventive activities included regular monitoring of cholesterol level and regular monitoring of blood pressure

^e^Self-medication was assessed as the intake of prescription and over-the-counter medications during the last two days

^f^Self-perceived health was assessed by asking subjects to rate their health. Responses “excellent”, “very good” and “good” were categorized as Good Health, while “mediocre” and “poor” were categorized as Poor Health

^g^i.e. mental disorders, diabetes, respiratory and cardiovascular problems

Age was stratified in four groups: 15–44, 45–64, 65–74, and ≥ 75 years. Employment status was categorized as Employed, Unemployed, Unpaid Work, Retiree or Unemployed for Medical Reasons, and Others. Socioeconomic status was determined based on the occupation of the household head (i.e., the person with highest income and/or employment status), and classified according to the Spanish adaptation of the British Registrar General’s scale into three categories [26, 27]: High, including Groups I and II (managers of public administrations and businesses; professions associated with university education; artists and sportsmen); Middle, including Groups III and IVa (civil servants, clerks and financial workers; self-employed; supervisors of manual workers and qualified manual workers); and Low, including Groups IVb and V (semi-qualified and non-qualified manual workers). Drinking behavior was categorized according to the subject’s alcohol consumption habits as Non-Drinkers (subjects who have not consumed alcohol in the last 12 months), Light Drinkers, and Heavy Drinkers (men who drink more than 28 standard drinks per week, women who drink more than 17 standard drinks per week, or subjects who drink more than five consecutive alcoholic beverages once a month, regardless of sex). (In Spain, one standard drink contains 10 g of ethanol.) [28] Physical activity was categorized as Healthy and Unhealthy according to an adaptation of the International Physical Activity Questionnaire (IPAQ) short form: the category “healthy” encompassed the IPAQ categories “moderate” and “high physical activity”, whereas the category unhealthy corresponded to the category “low physical activity” of the IPAQ [25, 29]. Preventive activities included regular monitoring of cholesterol level and regular monitoring of blood pressure. Self-medication was assessed as the intake of prescription and over-the-counter medications during the last two days. In subjects between 18 and 74 years old, body weight was measured using the Body Mass Index (BMI) based on subject-reported height and weight, and categorized as Underweight (BMI < 18.5 kg/m^2^), Normal Weight (≥18.5 to < 25 kg/m^2^), Overweight (≥25 to < 30 kg/m^2^) and Obesity (≥30 kg/m^2^). Self-perceived health was assessed by asking subjects to rate their health. Responses “excellent”, “very good” and “good” were categorized as Good Health, while “mediocre” and “poor” were categorized as Poor Health. Visits to healthcare professionals included visits in the last 12 months to general practitioners, sexual and reproductive healthcare professionals, mental health professionals, and other specialists. They also included visits to nurses, social workers, pharmacists and other community health agents. Subjects were also asked about their chronic disorders, their comorbidities (i.e., mental disorders, diabetes, and respiratory and cardiovascular problems), and their physical limitations to perform daily activities.

In 2014, ESCA included the shortened version of the European Health Literacy Questionnaire (HLS-EU-Q16), which contains 16 statements to measure HL in the general populations [14]. The statements in the questionnaire cover three different HL domains, i.e. Health Care, Disease Prevention, and Health Promotion. Each respondent was asked to give their opinion on a 5-point Likert scale: “very easy”, “easy”, “difficult”, “very difficult”, and “I don’t know”. Responses were dichotomized, with “very easy” and “easy” given a score of 1, and “difficult” and “very difficult” given a score of 0. Statements that were answered as “I don’t know” were treated as missing data in the analysis. Only participants who answered at least 14 out of all 16 items were included. HL was categorized in three levels: Sufficient (13–16), Problematic (9–12), and Inadequate (1–8). The HLS-EU-Q16 questionnaire has been validated in Spanish with a Cronbach’s alpha of 0.982 [24].

### Statistical analyses

To correct oversampling of less populated areas, an appropriate weight adjustment was applied before the analysis [25, 30, 31]. Continuous variables were summarized as means and their standard deviation (SD), and categorical variables were displayed as frequencies and percentages. To investigate the determinants of HL, the HLS-EU-Q16 index was dichotomized into two categories: Inadequate (inadequate and problematic), and Adequate (sufficient) HL [32].

Chi-square tests were performed to compare the percentages of subjects with adequate or inadequate HL across sociodemographic and health-related variables. For variables “smoking” and “alcohol drinking”, post hoc analyses were performed and the Bonferroni correction was used for adjusting the significance threshold. Variables showing differences with *p*-value < 0.1 in the univariate analysis were included in a stepwise logistic regression to predict inadequate HL level. Results were presented as OR and its 95% CI. For all other analyses, the significant threshold was set at a bilateral alpha level of 0.05. Receiver operating characteristic (ROC) curves were calculated to investigate the sensitivity and specificity of the HLS-EU-Q16 to predict inadequate HL. Univariate analyses were performed using the SPSS Statistics Package (IBM Corp. Released 2011. IBM SPSS Statistics for Windows, Version 20.0. Armonk, NY: IBM Corp), whereas the multivariate analysis and ROC curves were performed with an R package (R Foundation for Statistical Computing, Vienna, Austria).

## Results

### Overview of HLS-EU-Q16 results

**Fig. 1:**
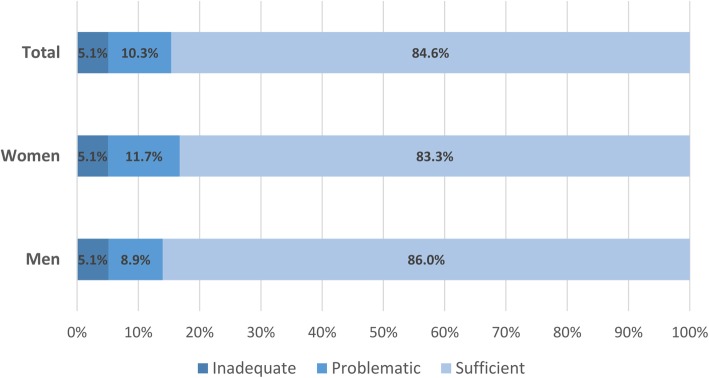
Levels of health literacy index by gender and for the total sample. HLS-EU-Q16 index: (0–8) Inadequate, (9–12) Problematic, and (12–16) Sufficient

The ESCA survey was administered to 3642 subjects. Of them, 2433 (70%), aged between 15 and 98 years old (mean of 45.9 years, SD 18.0), responded to the 16 items of HLS-EU-Q16 and were, therefore, considered for the analysis. Table 2 summarizes the responses to each item of HLS-EU-Q16. Overall, 2059 subjects (84.6%) showed sufficient HL, 250 (10.3%) inadequate HL, and 124 (5.1%) problematic HL, with no significant differences between men and women (*p* = 0.070) (Fig. 1).

**Table 2:** Percentage distribution of responses for HLS-EU-Q16 items

On a scale from very easy to very difficult, how easy would you say it is to …?		Very easy + easy	Difficult + very difficult	Don’t know
1	Find information on treatment of illnesses that concern you	77.6	15.6	6.8
2	Find out where to get professional help when you are ill	85.6	10.9	3.5
3	Understand what your doctor tells you	93.9	5.5	0.7
4	Understand your doctor or pharmacist’s instructions on how to take a prescribed medicine	96.2	3.2	0.6
5	Judge when you may need to get a second opinion from another doctor	69.8	16.7	13.5
6	Use information the doctor gives you to make decisions about your illness	77.3	15	7.7
7	Follow instructions from your doctor or pharmacist	96.4	2.5	1.1
8	Find information on how to manage mental health problems like stress and depression	70.1	16.6	13.3
9	Understand health warnings about behaviour such as smoking, low physical activity and drinking too much	94	4.1	1.9
10	Understand why you need health screenings	94.1	3.8	2.2
11	Judge if the information on health risks in the media is reliable	78	15.2	6.8
12	Decide how you can protect yourself from illness based on information in the media	82.5	12.5	5
13	Find out about activities that are good for your mental well-being	88.9	7.3	3.8
14	Understand advice on health from family members or friends	94.1	4	1.9
15	Understand information in the media on how to become healthier	88.9	7.7	3.4
16	Judge which everyday behavior is related to your health	93.5	4.5	2

### Subject characteristics and health behavior

When analyzing the distribution of HL, stratified as Sufficient and Inadequate or Problematic, by the sociodemographic characteristics of the study subjects, gender differences remained non-significant (Table 3). Conversely, the distribution of subjects across the categories of socioeconomic status, employment status, and study level revealed a significant trend towards being employed and belonging to a high socioeconomic class for subjects with sufficient literacy. Similarly, the percentage of individuals with secondary and university education level tended to be higher among subjects with sufficient literacy. Finally, subjects with sufficient HL were significantly younger than those with inadequate or problematic HL (Table 3).

**Table 3:** Health literacy according to socio-demographic characteristics of study subjects

	Overall	Health literacy		*P* ^a^
		Inadequate/problematic	Sufficient	
	(*N* = 2433)	(*N* = 374)	(*N* = 2059)	
Gender, No. (%)				
Men	1209 (49.7)	169 (45.2)	1040 (50.5)	0,058
Women	1224 (50.3)	205 (54.8)	1019 (49.5)	
Age (years), mean (SD)	45.9 (18.0)	54.3 (21.3)	44.4 (17.0)	< 0.001
Age groups, No. (%)				
15–44 years	1264 (52.0)	145 (38.8)	1119 (54.3)	< 0.001
45–64 years	750 (30.8)	94 (25.1)	656 (31.9)	
65–74 years	216 (8.9)	49 (13.1)	167 (8.1)	
≥ 75 years	203 (8.3)	86 (23.0)	117 (5.7)	
Employement status, No. (%)				
Employed	1313 (54.1)	145 (39.0)	1168 (56.8)	< 0.001
Unemployed	269 (11.1)	29 (7.8)	240 (11.7)	
Unpaid work	406 (16.7)	78 (21.0)	328 (15.9)	
Retired/Permanently disabled	432 (17.8)	117 (31.5)	315 (15.3)	
Other	9 (0.4)	3 (0.8)	6 (0.3)	
Socioeconomic status^b^				
High (groups I and II)	537 (22.1)	42 (11.2)	495 (24.0)	< 0.001
Middle (groups III and IVa)	496 (20.4)	57 (15.2)	439 (21.3)	
Low (groups IVb and V)	1341 (55.1)	258 (69.0)	1083 (52.6)	
NA (Household head has never worked)	39 (1.6)	14 (3.7)	25 (1.2)	
NR	20 (0.8)	3 (0.8)	17 (0.8)	
Education, No. (%)				
Primary or non-regulated education	352 (14.5)	121 (32.4)	231 (11.2)	< 0.001
Secondary education	1515 (62.3)	206 (55.1)	1309 (63.6)	
College/University/Post-graduate	566 (23.3)	47 (12.6)	519 (25.2)	

*NA* Not applicable, *NR* No response

^a^ Differences between inadequate/problematic and sufficient HL, assessed with Chi-square tests

^b^ Socioeconomic status was determined based on occupation the household head classified according to the Spanish adaptation of the British Registrar General’s classification [26, 27]

To investigate the relationship between HL and awareness and interest on health, variables related with health behavior were analyzed (Table 4). Drinking behavior, categorized as non-drinkers, light drinkers, and heavy drinkers, was significantly associated with HL level, with subjects in the sufficient HL group more frequently reporting light consumption. Healthy physical activity was more frequently reported in the group with sufficient literacy. The percentage of subjects who performed preventive activities, such as monitoring their blood pressure and cholesterol level, was significantly higher in the inadequate or problematic HL group. Subjects with inadequate/problematic HL also showed a significant trend towards self-medication and consumption of diet and homeopathic products, although differences with sufficient HL subjects were mild. A detailed analysis of the types of medication taken in the last two days revealed significant differences only in the consumption of anti-inflammatory and similar drugs (7.7 and 92.3% of subjects with inadequate/problematic and sufficient HL, respectively; *p* <  0.001).

**Table 4:** Health literacy according to the health behaviours of study subjects

	Overall	Health literacy		P*
		Inadequate/problematic	Sufficient	
	(*N* = 2433)	(*N* = 374)	(*N* = 2059)	
Smoking				
Smoker^a^	647 (26.6)	81 (21.7)	566 (27.5)	0.038
Former smoker^a^	499 (20.5)	75 (20.1)	424 (20.6)	
Non-smoker	1287 (52.9)	218 (58.3)	1069 (51.9)	
Alcohol consumption				
Non-drinker	838 (34.4)	184 (49.2)	654 (31.8)	< 0.001
Light drinker^b^	1476 (60.7)	174 (46.5)	1302 (63.2)	
Heavy drinker^b^	119 (4.9)	16 (4.3)	103 (5.0)	
Physical activity				
Healthy	1493 (69.7)	159 (61.9)	1334 (70.8)	0.003
Non-healthy	648 (30.3)	98 (38.1)	550 (29.2)	
Health coverage				
Only public health coverage	1764 (72.6)	297 (79.4)	1467 (71.4)	0.02
Public and private health coverage	658 (27.1)	77 (20.6)	581 (28.3)	
Only private health coverage	3 (0.1)	–	3 (0.1)	
No health insurance	4 (0.2)	–	4 (0.2)	
Preventive activities	1498 (61.6)	281 (75.1)	1217 (59.1)	< 0.001
Self-medication	294 (12.1)	261 (12.7)	33 (8.8)	0.035
Self-consumption of dietary/ homeopathic products	72 (3.0)	17 (4.5)	55 (2.7)	0.049

*Differences between inadequate/problematic and sufficient HL, assessed with Chi-square tests

^a^Comparisons between non-smoker category (significance level using Bonferroni correction *p* <  0.008)

^b^Comparisons between non-drinker category (significance level using Bonferroni correction *p* <  0.008)

Differences in HL level were also analyzed regarding variables related to the subjects’ health status (Table 5). Subjects with inadequate or problematic HL had higher BMI, and the percentage of subjects with normal weight was higher for those with sufficient level of literacy. The percentage of patients with good self-perceived health was also higher in subjects with sufficient HL. Conversely, subjects with inadequate/problematic HL tended to visit healthcare professionals more frequently, had a self-perceived chronic disorder and reported having comorbidities.

**Table 5:** Health literacy according to the subject-perceived health status

	Overall	Health literacy		*P* ^a^
		Inadequate/problematic	Sufficient	
	(*N* = 2433)	(*N* = 374)	(*N* = 2059)	
BMI (Kg/m^2^), mean (SD)	25.5 (4.5)	26.2 (5.1)	25.1 (4.3)	< 0.001
Obesity				
Underweight (< 18.5)	67 (2.8)	10 (2.7)	57 (2.8)	< 0.001
Normal Weight (≥18.5 a < 25)	1213 (50.2)	155 (42.2)	1058 (51.6)	
Overweight (≥25 a < 30)	809 (33.5)	128 (34.9)	681 (33.2)	
Obese (≥30)	329 (13.6)	74 (20.2)	255 (12.4)	
Perceived general health				
Good health	2029 (83.4)	259 (69.3)	1770 (86.0)	< 0.001
Bad health	404 (16.6)	115 (30.7)	289 (14.0)	
Visits to the healthcare professional				
Family doctor	1797 (73.9)	306 (81.8)	1491 (72.4)	< 0.001
Sexual and reproductive healthcare professional	553 (45.3)	61 (29.9)	492 (48.4)	< 0.001
Mental health professional	432 (17.8)	87 (23.3)	345 (16.8)	0.002
Other specialists	1613 (66.3)	236 (63.1)	1377 (66.9)	0.155
Nurse	302 (12.4)	74 (19.8)	228 (11.1)	< 0.001
Social worker	30 (1.2)	14 (3.7)	16 (0.8)	< 0.001
Pharmacists and other community health agents	489 (20.1)	54 (14.4)	435 (21.1)	0.003
Medical consultation last year (at least once)	2182 (89.7)	340 (90.9)	1842 (89.5)	0.397
Self-perceived chronic disorder	958 (39.4)	210 (56.1)	748 (36.3)	< 0.001
Comorbidities	1802 (74.1)	313 (83.7)	1489 (72.3)	< 0.001
Mental health disorders	385 (15.8)	83 (22.2)	302 (14.7)	< 0.001
Physical limitation to perform everyday activities				
Yes, severely limited	51 (2.1)	23 (6.1)	28 (1.4)	< 0.001
Yes, limited but not severely	183 (7.5)	56 (15.0)	127 (6.2)	
Not limited	2199 (90.4)	295 (78.9)	1904 (92.5)	

*BMI* body mass index

^a^ Differences between inadequate/problematic and sufficient HL, assessed with Chi-square tests

### Prediction models of health literacy (HL)

A logistic regression analysis to predict the relative association of specific variables with HL showed that a lower level of education and low socioeconomic status are associated with low HL (OR 2.08, CI 95% 1.32–3.28, *p =* 0.002 for primary education, and OR 2.11, CI 95% 1.42–3.15, *p* <  0.001 for low socioeconomic status). A physical limitation to perform everyday activities was also associated with low HL (OR 2.50, CI 95% 1.34–4.66, *p* = 0.004 for those severely limited). A more modest association was found with low physical activity, having a self-perceived chronic disorder, and performing preventive activities, such as monitoring their blood pressure and cholesterol level (Table 6).

**Table 6:** Multivariate logistic regression analysis to predict the probability of inadequate/problematic level of health literacy

Variable	Multivariate^a,b^		
	OR	CI 95%	*P*
Education ^c^			< 0.001
Primary or non-regulated education	2.08	1.32–3.28	0.002
Secondary education	1.13	0.77–1.64	0.54
College/University/Post-graduate^d^	1	–	–
Socioeconomic status ^e, f^			0.002
High (groups I and II) ^d^	1	–	–
Middle (groups III and IVa)	1.39	0.88–2.18	0.16
Low (groups IVb and V)	2.11	1.42–3.15	< 0.001
NA (Household head has never worked)	2.01	0.82–4.91	0.13
Physical activity			
Healthy ^d^	1	–	–
Non-healthy	1.40	1.06–1.85	0.017
Preventive activities			
Yes	1.36	1.03–1.80	0.029
No ^d^	1	–	–
Self-perceived chronic disorder			
Yes	1.31	1.01–1.70	0.046
No ^d^	1	–	–
Physical limitation to perform everyday activities ^g^			0.001
Yes, severely limited	2.50	1.34–4.66	0.004
Yes, limited but not severely	1.72	1.17–2.53	0.006
Not limited ^d^	1	–	–

*NA* Not applicable

^a^ Hosmer-Lemeshow test: *p* = 0.52

^b^ Probability of inadequate or problematic level of literacy = Exp(β) / (1 + Exp(β)), on β = −3.159 + 0.733 (in case of primary studies or without studies) + 0.119 (in case of secondary studies) + 0.326 (in case of middle socioeconomic status) + 0.748 (in case of low socioeconomic status) + 0.698 (in case of not applicable socioeconomic status (Household head has never worked)) + 0.339 (in case of unhealthy physical activity) + 0.311 (in case of performing preventive activities) + 0.266 (in case of self-perceived chronic disorder) + 0.914 (in case of severely limited to perform daily activities) + 0.542 (in case of limited to perform daily activities but not severely)

^c^ *p* value corresponds to the differences between the three groups (Primary or no studies; Secondary; or University)

^d^ Reference category

^e^ Socioeconomic status was determined based on occupation the household head classified according to the Spanish adaptation of the British Registrar General’s classification [25, 26]

^f^ *p* value corresponds to the differences between the four groups (High (groups I and II); Middle (groups III and IVa); Low (groups IVb and V); NA (Household head never worked))

^g^ *p* value corresponds to the differences between the three groups (Yes, severely limited; Yes, limited but not severely; or Not limited)

**Fig. 2:**
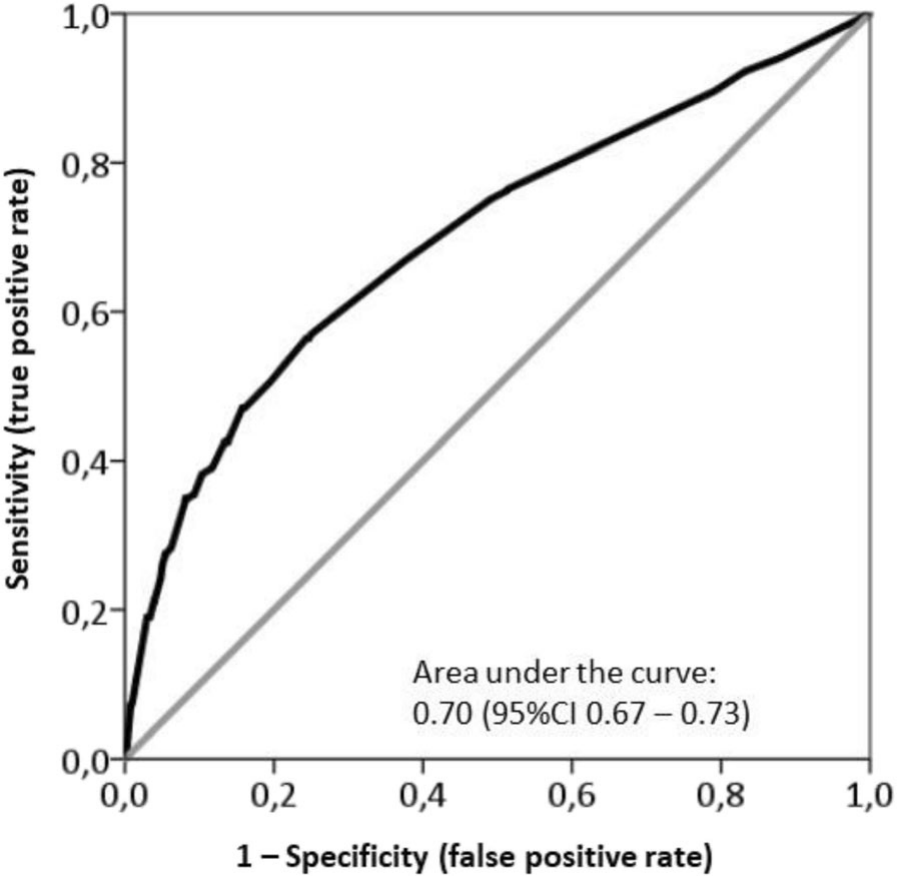
ROC curve for inadequate or problematic level of health literacy

The predictive capacity of the multivariate logistic regression model for the probability of inadequate or problematic level of literacy was analyzed through a ROC curve. The area under the ROC curve was 0.70 (CI 95% 0.67–0.73), which is an acceptable level of discrimination according to Hosmer et al. [33] (Fig. 2).

## Discussion

In this study, we benefited from a governmental health survey to investigate the HL level on a sample of 2433 subjects, randomly selected from a 7.5-million-people area. Our analysis revealed that 85% of people in this area have a sufficient HL level. Based on information regarding sociodemographic characteristics, health status, and health behavior in this population, the level of education, socioeconomic status, physical limitations to perform everyday activities, physical activity, performing preventive activities and self-perceived chronic disorders were identified as factors predicting an inadequate or problematic HL level. The low percentage of subjects with problematic or inadequate HL observed in this study (15.4%) stands out from previous studies investigating the HL level with the HLS-EU-Q16 Questionnaire, which reported percentages of inadequate/problematic HL ranging from 7 to 71% [12, 19, 23]. The wide variability in the percentages may be partially explained by the differences in study samples, which in some cases were selected from specific populations, such as Somali women living in Oslo [19], or subjects from a cohort study investigating cardiovascular risk [12]. The HLS-EU-Q16 Questionnaire has been recently validated in Valencia (a neighbor region in Spain with similar population than Catalonia); the percentage of subjects with problematic or inadequate HL (12.48%) were similar to our results [24].

Our results also differed from those observed by Sorensen et al., who used the full version of the questionnaire (HLS-EU-Q47) in a comparative study of the general population from various European countries, in which Spanish subjects showed one of the highest percentages of inadequate or problematic HL (58.3%), surpassed only by Bulgaria (62.1%) [14]. It is worth mentioning that in a preliminary validation of HLS-EU-Q16, in which the results of the 16-item and 47-item Questionnaires were investigated in various countries, Spain was the country with the lowest percentage of concurrent classification (67.5%), below the average of 75.8% [34]. Although the percentage of subjects with inadequate or problematic HL found by Pelikan et al. in the aforementioned study (34.4%) [34] is still far from that observed in our sample (15.4%) and in Valencia’s population (12.8%) [24], these inconsistencies might indicate a country-specific limitation of HLS-EU-Q16, which may affect the accuracy of the short questionnaire. It is worth mentioning, on the other hand, that the Cronbach’s alpha of the HLS-EU-Q16 in Valencia was 0.98, similar to the values for the HLS-EU-Q47 questionnaire in Europe (0.97) and in Spain (0.96) [24].

Owing to the low percentage of subjects with inadequate (5.1%) and problematic (10.3%) HL, we grouped these two categories to analyze factors potentially influencing HL. In our analysis, education level, socioeconomic status, physical activity, performing preventive activities, self-perceived chronic disorders, and having a physical limitation stood out as factors that significantly predicted an inadequate or problematic level of HL. According to the ROC curve, our model had an acceptable capacity to discriminate inadequate or problematic HL. The education level has been consistently reported as a factor associated with HL [12, 14, 23, 24, 35]. In 2013, the WHO considered people with low education level and/or low socioeconomic status as vulnerable groups with much higher proportions of limited HL than the general population in Europe [1]. The WHO also included those with worse health (status measured by self-perceived health, long-term illness and physical limitations to perform everyday activities due to health problems) in the specific vulnerable groups with limited HL [1]. In our study, perceived general health had no contribution to the multivariate model, but the perception of chronic disorders and —more importantly— physical limitations to perform everyday activities increased the odds of inadequate/problematic HL. Surprisingly, we also found that performing preventive activities, like monitoring cholesterol level and blood pressure, also increased the odds of low HL. Although further studies should be conducted to explain this unexpected relationship, the fact that people who periodically monitor their blood pressure and cholesterol level are often older people who deal with chronic diseases may have contributed to this finding. In this regard, the higher risk for low HL might lay on the underlying subject profile rather than the isolated feature of performing preventive activities. Finally, physical activity, which significantly contributed to our multivariate model, had been considered by the WHO as one of the health-related behaviors most strongly associated with HL [1]. However, Levin-Zamir et al. only found a borderline correlation between low HL and lack of physical activity, which was not significant in a multivariate analysis [23].

Obtaining an overview of HL among the population in our area and the incidence of social determinants in the level of literacy is mainstay for planning patient-centered healthcare services and policies. A deeper knowledge on HL in an area with a given sociodemographic profile may ultimately contribute to reducing inequalities in health through a “proportionate universalism”. The data show that people who are in vulnerable groups (for example, people with chronic diseases) have a lower level of literacy and, therefore, a greater need to improve their literacy level to be able to look after their health. Therefore, it is necessary that public authorities focus on the tailored needs of the patients and promote person-centered actions to improve the health competencies of these groups. For example, the expert patient program empowers chronic disease patients and their caregivers through peer-to-peer learning methods. Furthermore, it is also necessary to work on the promotion of tools that facilitate patients to make shared decisions about their health, which implies giving health information to the population, accessible, understandable, and with a salutogenic vision. Our results are strengthened by the fact that the survey was administered to a large number of subjects that were selected based on a multistage probability sampling, thus improving the reliability of the inference to the whole population. Furthermore, the survey was administered by trained professionals, who performed face-to-face home interviews. On the other hand, the study has some limitations that must be considered. First, although recommended by the WHO, questionnaire-based assessments of HL, like HLS-EU-Q16, do not include objective elements to measure functional HL and, therefore, can be associated with a reporting bias, particularly in variables regarding health behavior and health status [5, 14]. Moreover, respondents in face-to-face interviews tend to overrate their skills and underrate their problems [36]. Another limitation of our study was its cross-sectional design, which makes it difficult to draw causal inferences.

## Conclusions

Our study shows that Catalonia has a high percentage of subjects with sufficient HL. Despite the skewed distribution of our study sample across the various levels of HL, we could identify various determinants of HL, being the education level, socioeconomic status, and physical limitations the factors with the strongest contribution to problematic or inadequate HL. Our findings can aid policymakers focusing literacy programs to subjects who are at higher risk of low HL. Also, the high percentage of subjects with sufficient HL, along with the prevalence of some inadequate health behaviors, suggest that awareness-raising measures to improve popular health behaviors should take into account other determinants aside HL. Although these results are likely to be country-specific, the factors identified will allow policy makers of areas with similar socioeconomic profiles to identify patients or groups with high risk of problematic or inadequate HL, which is essential for a successful patient-centered model of care.

## Data Availability

The datasets used and/or analyzed during the current study are available from the corresponding author on reasonable request.

## Abbreviations

BMI: Body mass index
CATIC: Comissió Assessora per al Tractament de la Informació Confidencial (Consultants’ Committee of Confidential Information Management)
CI: Confidence interval
ESCA: Enquesta de salut de Catalunya (Health Survey for Catalonia)
HL: Health literacy
HLS-EU: European Health Literacy Survey
HLS-EU-Q16: European Health Literacy Survey Questionnaire (short version)
HLS-EU-Q47: European Health Literacy Survey Questionnaire (full version)
HPC: Health Plan for Catalonia
IPAQ: International Physical Activity Questionnaire
OR: Odds ratio
ROC: Receiver operating characteristic
SD: Standard deviation
WHO: World Health Organization
